# Audiovisual Training in Virtual Reality Improves Auditory Spatial Adaptation in Unilateral Hearing Loss Patients

**DOI:** 10.3390/jcm12062357

**Published:** 2023-03-17

**Authors:** Mariam Alzaher, Chiara Valzolgher, Grégoire Verdelet, Francesco Pavani, Alessandro Farnè, Pascal Barone, Mathieu Marx

**Affiliations:** 1 Research Center of Brain and Cognition, CerCo, CNRS, 31000 Toulouse, France; 2 ENT Department, University Hospital of Purpan, 31000 Toulouse, France; 3 Center for Mind/Brain Sciences—CIMeC, University of Trento, 38100 Trento, Italy; 4 Impact Team of the Lyon Neuroscience Research Centre INSERM U1028 CNRS UMR5292, University Claude Bernard Lyon I, 69000 Lyon, France; 5 Neuroimmersion, Lyon Neuroscience Research Center, 69000 Lyon, France; 6 Centro Interuniversitario di Ricerca “Cognizione, Linguaggio e Sordità”—CIRCLeS, University of Trento, 38100 Trento, Italy

**Keywords:** spatial adaptation, audiovisual training, virtual reality, unilateral hearing loss, head movements

## Abstract

Unilateral hearing loss (UHL) leads to an alteration of binaural cues resulting in a significant increment of spatial errors in the horizontal plane. In this study, nineteen patients with UHL were recruited and randomized in a cross-over design into two groups; a first group (*n* = 9) that received spatial audiovisual training in the first session and a non-spatial audiovisual training in the second session (2 to 4 weeks after the first session). A second group (*n* = 10) received the same training in the opposite order (non-spatial and then spatial). A sound localization test using head-pointing (LOCATEST) was completed prior to and following each training session. The results showed a significant decrease in head-pointing localization errors after spatial training for group 1 (24.85° ± 15.8° vs. 16.17° ± 11.28°; *p* < 0.001). The number of head movements during the spatial training for the 19 participants did not change (*p* = 0.79); nonetheless, the hand-pointing errors and reaction times significantly decreased at the end of the spatial training (*p* < 0.001). This study suggests that audiovisual spatial training can improve and induce spatial adaptation to a monaural deficit through the optimization of effective head movements. Virtual reality systems are relevant tools that can be used in clinics to develop training programs for patients with hearing impairments.

## 1. Introduction

One of the fundamental functions of binaural integration is sound source localization and the perception of targets in the presence of competing noise [1]. In the case of acquired unilateral hearing loss (UHL), the symmetrical integration of interaural time and intensity differences is severely altered, leading to a significant disruption in spatial hearing. Despite the spatial deficit, there is growing evidence that compensation after UHL takes place due to the plasticity of central auditory processing and an adaptation and recovery in spatial auditory performance [2,3]. Animal studies that examined the effect of UHL on cortical activation suggest a possible weakening of the neural representation of the deaf ear accompanied by a strengthening of the neural representation of the opposite intact ear in monaural listening situations. This pronounced reorganization in favor of the better ear in the case of asymmetrical hearing can also be observed in unilateral cochlear implantation after profound symmetrical deafness in cats [4] and in children [5]. Parallel to neural adaptation, different studies tried to understand the behavioral changes that accompany monaural hearing conditions [6]. They suggest that listeners with UHL learned to make use of the spectral shape cues due to the direction-dependent filtering of the pinna in the intact ear to better judge sound direction. The important role of monaural cues was shown, for example, in experiments with pinna shape modification that led to an increase in localization errors [7]. Experiments on monaural plugging in ferrets showed that spatial adaptation took place, and that is presumably related to the use of the spectral cues of the better ear [8]. In humans, Slattery and Middlebrooks (1994) propose that the use of these spectral cues can be learned and enhanced in some patients with UHL [6]. In addition to spectral pinna cues, the subsequent work of Hendrikse et al. and Friedman et al. [9,10] noted an important implication of the head shadow effect (HSE) in sound localization. The HSE interferes with the perception of sound intensity; in a free field, a stimulation is filtered and attenuated, especially in the azimuth, due to the acoustic properties of the head; therefore, extracting horizontal spatial information from intensity analyses is possible. However, it is unknown to what extent the HSE can play a role in detecting accurate azimuth sound sources [9].

It has also been shown that the reliance on both the HSE and monaural cues can be enhanced in active listening, i.e., localization using head movements [11]. The first to document the importance of head rotation in reducing spatial ambiguities and front–back confusions was Wallach in 1940. Recent studies using virtual reality and motion tracking confirmed that sound localization accuracy could be improved with the use of head movements [12,13,14,15]. These studies suggested that modifying interaural cues using head movements produces a change in the angle specified initially by these cues, and the dynamic change of this angle during active listening can disambiguate spatial information and enable spatial discrimination and localization of a single sound source [9]. All these factors can be helpful for patients with UHL to adapt to their monaural hearing conditions. Middlebrooks, in 1994, showed that despite the binaural deficit, some patients manage to surpass the spatial deficit and present near-normal spatial accuracy in the horizontal plane [6]. Although the experimental procedure of Middlebrooks, 1994, did not provide additional information on the potential mechanisms involved in monaural adaptation, it seems that the factors cited above could have variable interindividual contributions.

Visual integration was found to have a significant impact on spatial auditory functions. In the animal model, for example, studies on owls reared with binocular prisms placed over their eyes to displace the visual field from the first day of eye-opening, without any change in their acoustic cues, revealed a spatial auditory shift towards the direction of the altered visual representation field [12]. In the case of altered binaural cues, studies on ferrets showed rapid spatial recovery due to audiovisual training [13]. In humans, the introduction of minimal visual cues in a virtual reality task changes spatial auditory performance in normal-hearing participants [14]. In the case of monaural listening, audiovisual spatial training improved sound localization even in an auditory-only condition [15]. These findings indicate that the possible adaptation to monaural hearing outside the training programs can be enhanced and accelerated if a rigorous and regular audiovisual training program are applied.

Although it remains unclear how UHL patients adapt to monaural hearing, the growing evidence mentioned above shows that these compensatory mechanisms must be studied in a testing procedure able to control all the features that can interfere with spatial auditory behavior. To date, the majority of experimental designs used to evaluate spatial functions do not have full control over the possible factors involved in spatial auditory perception. Classical methods usually evaluate spatial hearing in one dimension, with a limited number of speakers and thus limited angular positions. In addition to weak control of the possible effects of the visual environment and visual references during the experiment, the control of the implication of head movements (the number and reaction times) remains rare. A recent study by Valzolgher et al. [14] was able to validate the utility of a virtual reality (VR) tool using a head-mounted display (HMD_VIVE) in assessing spatial behavior. Notably, the VR system was able to track head movement behavior, including the angle of movements, reaction times, and the number of movements. Additionally, the VR system can provide the participant with an immersive experience where different conditions can be easily manipulated and tested (visual and non-visual).

In the present study, we aimed to explore the behavioral mechanisms adopted by adults with UHL to compensate for their binaural deficit. Our main objective was to assess the efficacy of the spatial training program on the localization abilities of participants with unilateral moderate to profound hearing loss, as has already been performed in previous studies on normal-hearing participants with monaural plugs [16] and bilateral cochlear implants [17]. We also aimed to decipher the adaptive behavioral mechanisms used to improve spatial skills by UHL participants. The present study can be an open door for future clinical implications of the VR approach in training programs that provide extensive and rapid adaptation to UHL.

## 2. Materials and Methods

### 2.1. Population

**Figure 1:**
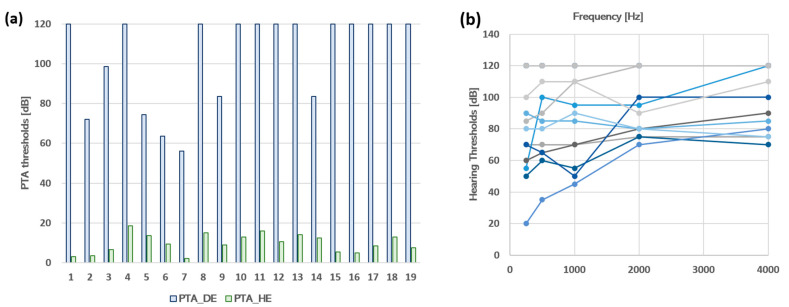
(**a**) Levels of hearing loss for the 19 unilateral hearing loss patients. The green histogram bars represent the hearing level in the healthy ears of the patients. The blue histogram bars represent the hearing level in the deaf ears of the patients. Abbreviations: PTA_HE = ‘Pure Tone Audiometry in the Healthy Ear’; PTA_DE = ’Pure Tone Audiometry in the Deaf Ear’. (**b**) Individual hearing thresholds. The hearing thresholds are presented for the deaf ear of each patient at the tested frequencies (250, 500, 1000, 2000, and 4000 Hz).

Twenty patients with unilateral moderate to profound hearing loss (Age = Mean ± SD, 51.4 ± 11.8, 10 right deafness, and 12 women) were recruited from the Ear, Nose, and Throat Department of Toulouse University Hospital. One patient did not complete the protocol and was eliminated from the study. The experiments took place in the Brain and Cognition Research Center in Toulouse (CerCo), France. All participants underwent an audiometric test to verify their hearing levels at the following frequencies, 250, 500, 1000, 2000, and 4000 Hz for both ears separately. The average of pure tone audiometry for each patient at each ear is presented in Figure 1, and the age, gender, and side and etiology of deafness of the 19 participants are presented in Table 1.

### 2.2. Experimental Sessions

The protocol comprised two sessions separated by a washout phase that lasted two to four weeks. In each session, patients underwent a pre- and post-test that we will call the LOCATEST and one training type (spatial training or non-spatial training). The order of the training types was counterbalanced between the 19 participants in a within-subject cross-over design. Nine participants underwent spatial training in the first session and non-spatial training in the second session (group 1). Ten participants performed the opposite order of the sessions, with non-spatial training in the first session and spatial training in the second session (group 2). There was no significant difference between group 1 and group 2 in terms of hearing loss thresholds in the deaf ear (104.33 ± 24.03 vs. 103.25 ± 23.21; *p* = 0.92) and in terms of age (52.56 ± 14.51 vs. 51.50 ± 8.32; *p* = 0.86).

### 2.3. Description of the VR material

Virtual reality and kinematic tracking were implemented using the HTC Vive (Vive Enterprise). The VR system used for this experiment was validated for experimental use in research [14]. The system comprises a head-mounted display (HMD, 1800 × 1200 px) with two base stations positioned at two opposite angles of the room with a distance of 4 m to detect position and motion of HTC Vive objects. The base stations continuously scanned and tracked the moving objects inside a play surface defined by the experimenter before the beginning of the experiment. The moving objects tracked by the base station in real-time were the HMD, the controller held by the patients in their hands during the training, and the tracker positioned above the speaker used to deliver the sound.

### 2.4. Experimental Procedure of Head-Pointing Localization Test (LOCATEST)

**Figure 2:**
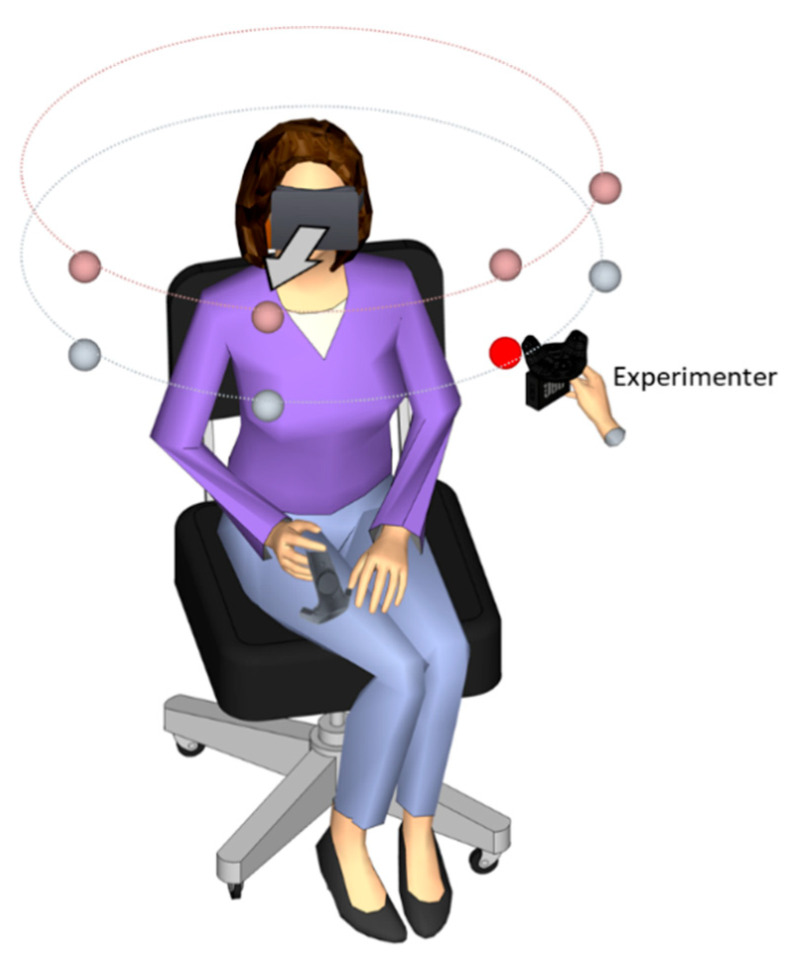
Experimental setup of head-pointing localization task (LOCATEST). The illustration shows the participant wearing the head-mounted display (HMD) during the LOCATEST. The eight spheres indicate predetermined speaker positions, which were not visible in the HMD. The hand holding the speaker near the red sphere was the experimenter’s hand, who moved the speaker to reach the target position.

The first test was a sound localization task using the head-pointing method. The participants sat in the center of the room on a rotating armless chair with the HMD mounted on their heads. The participants were immersed in a room with green walls with the same dimensions as the real experimental room of the lab (2.65 m × 2.85 m, width = 4.90 m). The sound stimulation was delivered in a free field at four different azimuth positions (−67.5°, −22.5°, 22.5°, and 67.5°) and two elevation positions (+5° and −15°) at a constant distance of 55 cm from the center of the patient’s head. The experimenter positioned the speaker in each location by following instructions visible on a monitor. The sound consisted of a 3 s white noise burst and amplitude modulated at 2.5 Hz with an intensity of 65 dB at the participant’s head (for more details about the procedure, check [15,18]) (Figure 2).

Patients were asked to localize the emitted sound by pointing, with their heads, towards the sound direction. They were also informed that they were free to move their head in any direction and that the sound could come from any position in the 3D plane. At the beginning of each trial, a central fixation cross appeared in the HMD; the fixation cross helped the participants to easily point their heads towards the sound source using the cross as a visual reference of their movements. There were no additional visual cues added to the visual scene. After sound emission, patients were asked to direct their heads toward the sound source direction and to validate their head position (their answer) by clicking on a controller that was held in their right hand. The experimenter explained that the response should be carried out using the head-pointing only, with no hand-pointing using the hand controller, which would only be used for head position validation. The LOCATEST was completed after 40 trials and was applied twice in every session, before and after the spatial and the non-spatial training.

### 2.5. Experimental Procedure of Spatial Training

The spatial training took place in the same experimental room with the same experimental condition as the LOCATEST. When the participants wore the HMD, they could see a virtual array of 13 loudspeakers arranged in a semicircle spanning from −72° at the left to +72° at the right, with the circular array falling into the visual field of the participant to maximize the efficiency of the use of visual cues with a 12° angular separation between each speaker at a distance of 55 cm from the participants’ heads. Small angle separation was important to evaluate the efficiency of the audiovisual spatial training. The sound was randomly delivered through the 13 positions using two different types of sound stimulation. Half of the stimuli were amplitude modulated at 2 Hz, and the remaining half was modulated at 3 Hz, with a total of 156 stimuli at the end of the training.

**Figure 3:**
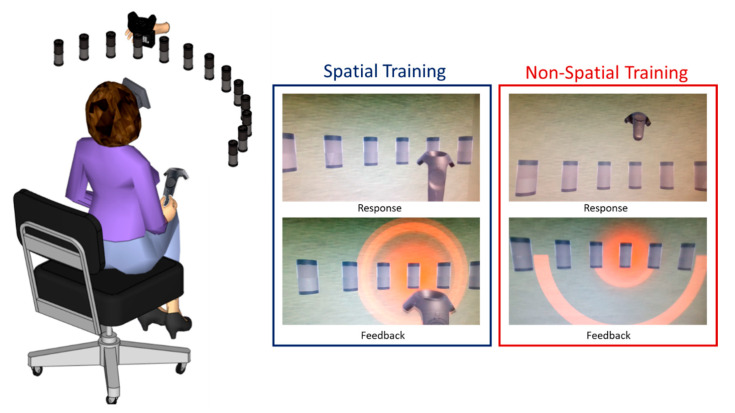
Experimental setup of both spatial and non-spatial trainings. In the spatial training, the participant was asked to judge the spatial location of the sound by touching the corresponding speaker with the controller held in their right hand. In the non-spatial training, the patient was asked to judge the quality of the sound by positioning the controller up if the sound was amplitude modulated at 3 Hz and down if the sound was amplitude modulated at 2 Hz. In both trainings, visual feedback was provided in the case of a wrong response.

The participants were holding a controller in their hands. They were asked to judge the spatial location of the emitted sound by touching the virtual speaker using the hand-held controller; participants were free to move their heads. The sound stimulation was continuous, and it only stopped when the participant touched the correct speaker. In the case where the participant touched the wrong speaker, the correct speaker would light up in red, and the participant was invited to correct their answers by touching the speaker lit up in red. Figure 3.

### 2.6. Experimental Procedure of Non-Spatial Training

The stimulation procedure of spatial and non-spatial trainings was the same. The only difference was that in the non-spatial training, patients were not asked to judge the spatial location of the sound; instead, they were asked to judge the sound quality (or amplitude modulation) by pointing their hand-held controller upwards (above the central speaker) if the stimulation sounded fast (amplitude modulation at 3 Hz) and downwards (below the central speaker) if the stimulation sounded slow (amplitude modulation at 2 Hz). If the participant reported an incorrect answer, for example, pointing upwards when the sound was amplitude modulated at 2 Hz, the central speaker would light up in red with an indication of the correct direction. Similarly to the spatial training, the stimulation only ended when the participant reported a correct answer (Figure 3).

### 2.7. Data Analysis

#### 2.7.1. Head-Pointing Localization Performances (LOCATEST)

In the LOCATEST, two types of variables were analyzed. (1) The azimuth head-pointing localization error (absolute error), designated by the angular distance between the speaker position and the patient’s head-pointing projection in the azimuth at the moment of the response validation. (2) The number of head movements during and after the sound emission. The head movements were manually selected by visualizing the velocity and the spatial rotation using a custom-made toolbox in MATLAB 2020. We fitted the absolute head-pointing localization error in the azimuth and the number of head movements in linear mixed-effect (LMER) models using the following statistical packages in R software (emmeans, lme4, and lmerTest).

#### 2.7.2. Performances during Audiovisual Training

For the spatial training, we decided to study the variation of the hand-pointing errors, the number of head movements, and the head reaction times across the 156 trials to check if there was an effect of the audiovisual spatial training on auditory spatial behavior within the training session. We averaged the hand-pointing localization errors committed at each trial by the 19 participants (the average of 19 responses by trials), and we evaluated the linear regression of the errors across the 156 trials. We performed the same procedure for the reaction times (sec) and the number of head movements.

All the data visualizations were plotted using the function ggplot in R studio 3 February 2022.

## 3. Results

### 3.1. Performances in Head-Pointing Localization (LOCATEST)

**Figure 4:**
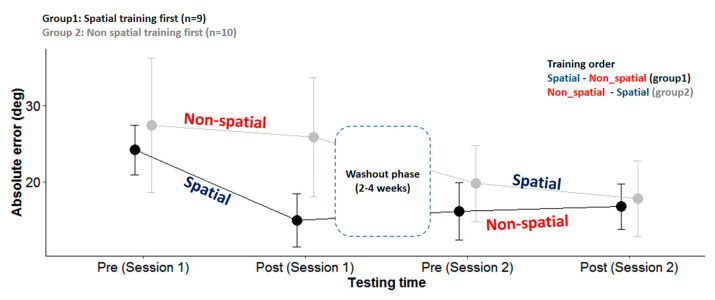
Head-pointing localization errors (LOCATEST) for each group pre- and post-spatial and non-spatial trainings. Session 1 designates the session on the first day. Session 2 designates the session on the second day, 2 to 4 weeks after the first session.

Before analyzing the data on the group level (*n* = 19), we decided to test the variation between each group separately according to the order of the session. We fitted the absolute horizontal errors collected during the LOCATEST in a linear mixed-effect model with the group (group 1 and group 2), session (day 1 or day 2), and phase (pre- and post-) as the fixed factors and the participants as the random factor (the intercept and slope). We found an effect of the phase (X^2^(1,N = 19) = 40.3, *p* < 0.001) and session (X^2^(1,N = 19) = 40.3, *p* < 0.001) but no effect of the group (*p* > 0.05). However, importantly, we found an interaction between the groups, session, and phase (X^2^(1,N = 19) = 4.46, *p* < 0.05). This interaction was induced by a significant reduction of errors in session 1 for group 1 but not for group 2 and a slight reduction in session 2 for group 2 but not for group 1, as illustrated in Figure 4. Group 1 (the spatial training first) showed a significant reduction of absolute azimuth errors in session 1 after the spatial training (24.85 ± 15.8 vs. 16.17 ± 11.28; *p* < 0.001). This spatial gain was maintained after the washout (pre-sessions 2: 16.05 ± 15.37) and was not influenced by the non-spatial training (post-session 2: 16.75 ± 13.46; *p* = 0.977), where the azimuth error remained low. For group 2 (the non-spatial training first), no significant change was noted after the non-spatial training during session 1 (27.37 ± 30.52 vs. 25.83 ± 26.42; *p* = 0.846). The horizontal errors were significantly reduced after the washout phase in session 2 (day 1, post-, 25.83 ± 26.42 vs. day 2, pre-, 19.75 ± 19.15; *p* < 0.05). The significant drop in the errors during the washout phase was driven by the performance of two patients who presented atypical performance compared to the 17 participants: patient number 19, who drastically reduced her errors from 74° (post-session 1) to 33° (pre-session 2), and patient number 15, who reduced his errors from 65° (post-session 1) to 56° (pre-session 2). The eight patients left in group 2 maintained a regular reduction of errors of 2.3° ± 5.6°, which is comparable to the reduction in group 1 (1.8° ± 3°); thus, after the spatial training was applied on the second day, the errors seemed to slightly reduce within the session for the whole of group 2 (*n* = 10); however, this reduction was not statistically significant (day 2, pre-, 19.75 ± 19.15 vs. day 2, post-, 17.78 ± 18.49; *p* = 0.772) (Figure 4).

**Figure 5:**
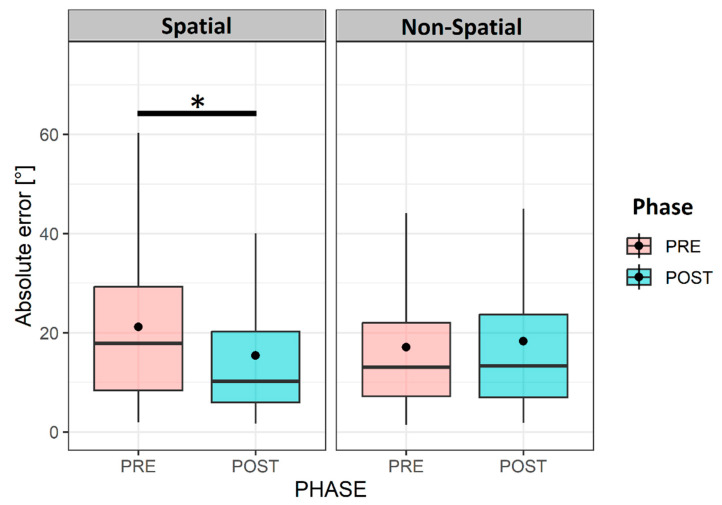
Horizontal errors’ variation according to training type and phase. Illustration of the variation of the absolute horizontal head-pointing errors in the LOCATEST, regardless of the order of the session in the 19 UHL patients. The asterisk (*) symbol in the legend denotes statistical significance at the alpha level of 0.05.

To study the impact of the spatial training on our 19 participants, we used a linear mixed-effect model with the training (spatial and non-spatial) and phase (pre- and post-) as the fixed factors and the participants and session as the random factors. The analysis showed an effect of the phase (X^2^(1,N = 19) =5.37; *p* < 0.05) and an interaction between the phase and training (X^2^(1,N = 19) = 4.28; *p* < 0.05) on head-pointing precision in the horizontal plane. The horizontal errors in the LOCATEST significantly decreased after the spatial training (pre-, 22.17 ± 17.8, vs. post-, 16.51 ± 15.54; *p* < 0.05), while no significant change was noted after the non-spatial training (pre-, 22.21 ± 24.6, vs. post-, 21.53 ± 21.7; *p* > 0.05), as shown in Figure 5.

**Figure 6:**
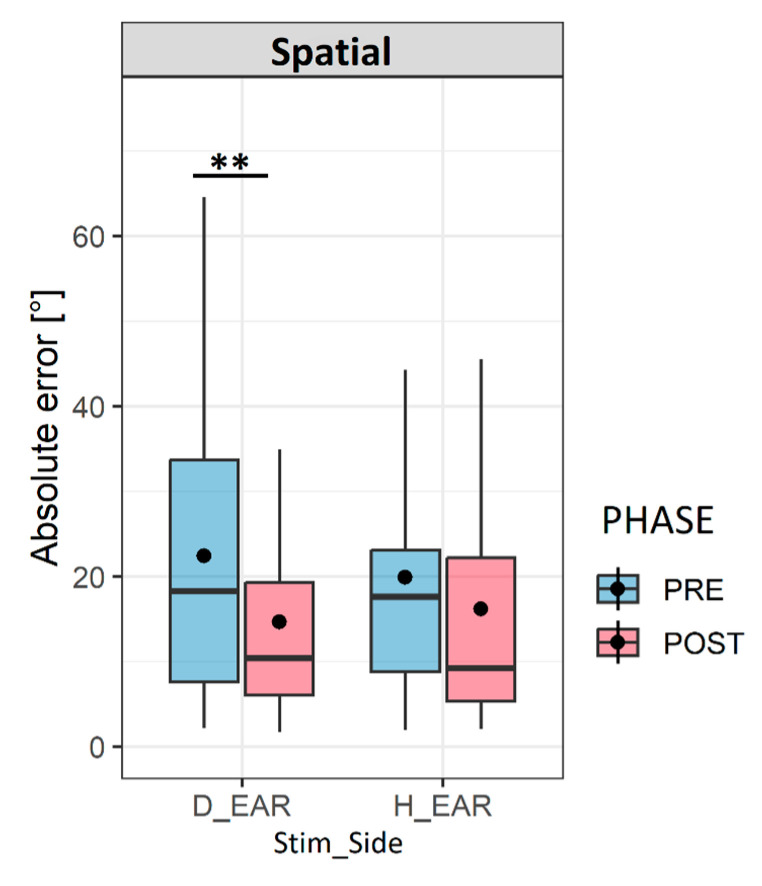
Variation of localization errors according to stimulation side. The box plots represent the variation of the horizontal absolute head-pointing error on the side of the deaf ear (D_EAR) and on the side of the healthy ear (H_ear) before (PRE) and after (POST). Abbreviations: Stim_Side: ‘stimulation side’; H error: ‘horizontal error’. The asterisk (**) symbol in the legend denotes statistical significance at the alpha level of 0.01.

We wanted to step further into the details of the spatial improvement after the audiovisual spatial training, and we decided to test the variation of the absolute horizontal error as a function of the stimulation side (the deaf and healthy ears); we noticed that the decrease in the horizontal errors after the spatial training was mainly on the side of the deaf ear. The errors significantly decreased from (24.99 ± 20.52) to (16.33 ± 16.30) (*p* < 0.05) on the side of the deaf ear, while no significant change was noted on the side of the healthy ear pre- (19.34 ± 14.18) and post- (16.7 ± 14.85) training (*p* > 0.05), as shown in Figure 6.

**Figure 7:**
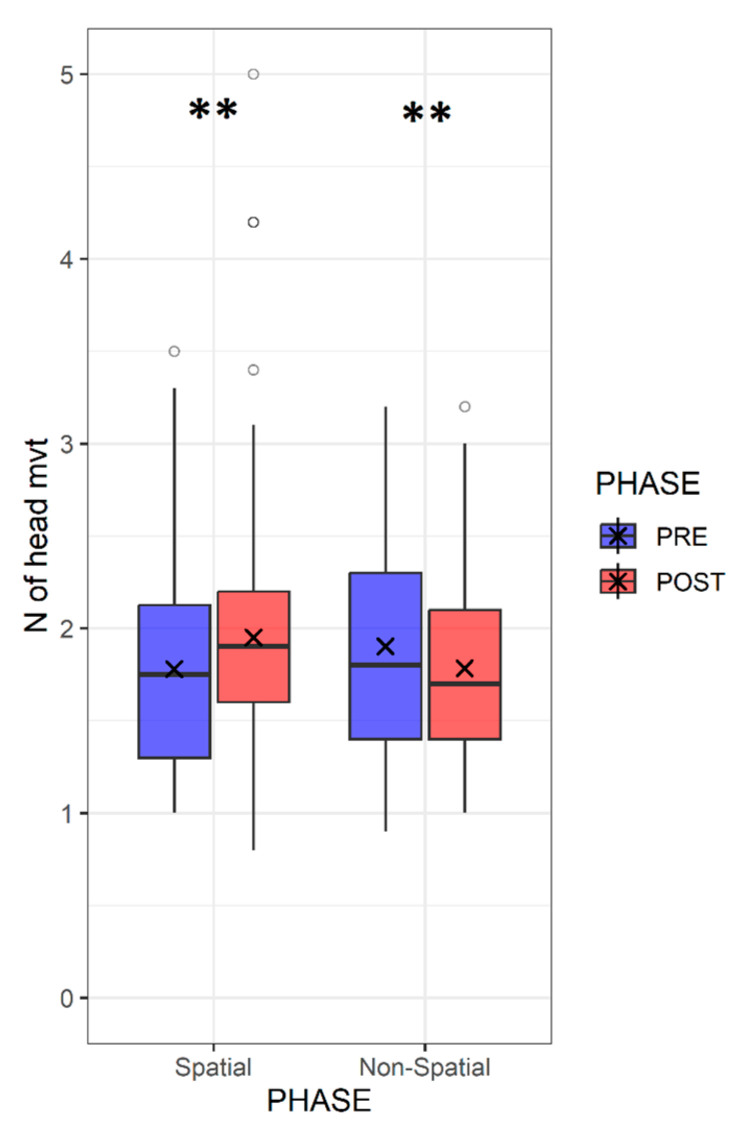
Variation of the number of head movements according to the training type. Box plots representing the variation of the number of head movements in the head-pointing task (LOCATEST) before and after the spatial and the non-spatial training for the 19 patients with UHL. The asterisk (**) symbol in the legend denotes statistical significance at the alpha level of 0.01.

In addition to the effect of the stimulation side, we assumed that head movements could also play an important role in guiding and orienting the participants. Therefore, we wanted to investigate the influence of the two training types on the number of head movements. Using the LME model, we analyzed the variation in the number of head movements as a function of the training and phase. We noticed that after the spatial training, the number of head movements increased during the task (pre-: 1.77 ± 0.52 vs. post-: 2.01 ± 0.83; *p* < 0.01), while this number decreased when the non-spatial training was applied (pre-: 1.9 ± 0.57 vs. post-: 1.78 ± 0.45; *p* < 0.01) (Figure 7).

### 3.2. Performance during Audiovisual Training

**Figure 8:**
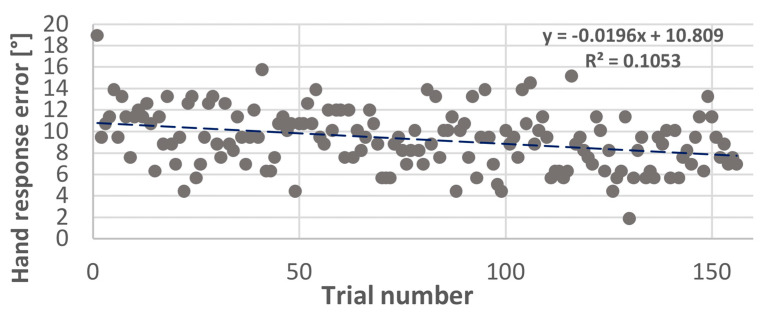
Variation of hand-pointing errors across trials. Scatter plot representing the horizontal errors of hand-pointing during the spatial training with 156 trials.

In the previous section, we showed that spatial training had an impact on both the spatial errors and the number of head movements (Figure 8). We wanted to step further into the strategies used during the spatial training. Therefore, we decided to study the behavioral changes across 156 trials during the spatial training. We studied the ability of the trial number to predict the hand-pointing errors, number of head movements, and head reaction time using a simple linear regression model. We found a significant relationship (*p* < 0.001) between the trial number and hand response errors (R² = 0.1053), and we also found a significant relationship (*p* < 0.001) between the trial number and head reaction time (R² = 0.0038); however, this relationship was not significant (*p* = 0.82) for the number of head movements, which did not change across 156 trials. The hand response errors were significantly reduced from (18.94° ± 32.81°) in the first trial to (6.94° ± 9.22°) in the 156th trial. Similarly, the head reaction time, designated by the first head movement during the stimulation, was significantly reduced (trial 1: 1.17 sec ± 0.41 and trial 156: 1.05 sec ± 0.58), while the number of head movements did not change (trial 1: 2.36 ± 1.11 and trial 156: 3 ± 1.76).

## 4. Discussion

### 4.1. Effect of Audiovisual Training on Spatial Hearing Abilities in UHL

The aim of this study was to evaluate the effectiveness of audiovisual spatial training on auditory spatial behavior displayed by UHL patients to compensate for their binaural deficit. The cross-modal approach in treating auditory deficits has indeed shown effectiveness in improving spatial perception [14,15]. Anatomical and electrophysiological studies in animal models showed the involvement of multisensory processing in the early stages of sensory perception, with this multisensory processing helping to re-calibrate unisensory modalities independently [19]. Anatomically, this can be explained by the presence of direct projections originating from the representation of the peripheral visual fields in the pre-striate cortex [20] towards the caudal areas of the auditory cortex, which are involved in spatial processing [21]. A large body of studies proved that multisensory stimulation that includes visual feedback is more effective than unisensory stimulation in normal-hearing participants with monaural plugs [15,16,18,22] and in patients with cochlear implants [17,23]. The study of [15] suggests that unisensory auditory training, applied to normal-hearing participants with ear plugs, showed no improvement in spatial behavior. However, when a visual cue was added, the horizontal errors decreased significantly on the side of the plugged ear. In the animal model, the seminal work of [24] proved the existence of a strong relationship between the width of the field of best vision and auditory spatial acuity, suggesting that the common behavior between mammals when hearing a sound is the motor reflex of eye orientation, followed by a head orientation to explore space.

In the present study, multisensory training played a crucial role in guiding vision towards the auditory stimulus, but it led to an improvement of spatial behavior even in the unisensory task of head-pointing without visual cues (LOCATEST). In the LOCATEST, where no visual cue can indicate the location of the sound, patients had to point with their heads toward the sound source. Despite their hearing loss level (the average hearing loss = 103 dB), these patients were able to improve their spatial localization after the audiovisual spatial training. This result is consistent with the findings of [16] on normal-hearing participants fitted with monaural plugs, where the same audiovisual spatial training improved spatial performance in the unisensory auditory spatial task by decreasing the degree of head-pointing localization errors from 19.3° before the spatial training to 11.5° after the training [16].

This effect of multisensory training on unisensory experience was also found in adult patients with bilateral cochlear implants (BCIs); a study by [22] showed faster improvement after multisensory spatial training compared to training in the auditory modality only. Although both populations (UHL and BCIs) have different adaptation strategies, they both rely on visual sensory inputs to optimize environmental spatial mapping [25]. Adding a new informative cue about sound location, such as vision in this case, is a helpful experience that minimizes listening effort and improves rapid learning [26].

### 4.2. Re-Learning to Move the Head Effectively

Spontaneous head movements are used in daily life to direct vision and localize visual and auditory objects [24]. Thus, the use of head movements by the listeners helps them to optimize the advantage of binaural cues by continuously changing the ITD (interaural time difference) and ILD (interaural intensity difference) [9]. The spontaneous reaction of turning the head and body towards a sound source has been found to be an important factor in weighing auditory cues [27,28]. Studies comparing static and active listening conditions showed better spatial performance when head movements were allowed [18,22,29].

In the case of UHL, binaural symmetry is disrupted, leading to a disruption in the auditory spatial acuity [28,30]. In this case, head rotations can play a strategic role in compensating and guiding spatial behavior by enhancing the head shadow effect and by guiding the head towards a sound position [31,32,33].

Most of the studies examining spatial auditory behavior in hearing-impaired participants tested spatial localization in conditions where the head was immobilized [28,34]. Nonetheless, this condition does not reflect the ecological situation, where listeners are continuously exploring their environment through the motor orientation of the eyes, head, and body.

Thus, studies that tended to assess spatial behavior in monaural listening with the head immobilized showed a significant increase in errors in the side of the plugged ear [6,31,32]. Head shadow effects can be more effective when moving the head by masking unnecessary noise and concentrating on the target sound [11]. This benefit is due to the spatial advantage extracted from the HSE, which is able to attenuate and filter sound due to the head’s properties, especially in azimuth. Varying sound intensity in the unplugged ear during head movements to optimize the head shadow advantage is an efficient provider of spatial information [33]. This improvement is noted in both horizontal and vertical planes [11]. This advantage explains the common behavior adopted by UHL patients to project their localization answers towards the side of the hearing ear [6]. The UHL patients displace their better ear towards the source location by rotating their head in order to increase the level of sound in the hearing ear and to reduce spatial ambiguities [35,36].

This “better ear preference” is also present in our UHL population, as shown in Figure 6. The advantage of our task in the LOCATEST is that we included head movements not only by allowing mobilization but also by using head-pointing as a localization task. In the head-pointing localization task, before the spatial training, localization errors were higher on the side of the deaf ear compared to the non-deaf ear (24.9° vs. 19.3°). Interestingly, the decrease in localization errors after the spatial training was on the side of the deaf ear only (from 24.9°(pre-) to 16.3° (post-)). In our study, the specific improvement of spatial accuracy on the side of the deaf ear can be explained by a “re-learning” to move the head effectively in order to optimize the advantage from the head shadow effect. Thus, when we analyzed head movement behavior during the audiovisual spatial training, we found no change in the number of head movements; however, the head reaction times and hand-pointing errors decreased at the end of the training. Even though the number of head movements did not change during the spatial training, patients used their head movements in a more effective manner.

The advantage of the task in the LOCATEST, as mentioned earlier, is that it forced the patient to use head movements in a unisensory task where no visual feedback was used as an orienting cue for the eyes and the head. We also used a stimulus duration of 3 s to make sure that dynamic tracking of the sound, which usually starts after 300 ms, was taking place [27,28]. Although the localization modality in the spatial training was different (hand-pointing vs. head-pointing in the LOCATEST), patients rapidly learned how to use their heads effectively. Valzolgher et al. [16] noted the same rapid adaptation in simulated monaural hearing, suggesting that participants learn to explore wider angles of the space after spatial training. The study of Valzolgher [16] also noted the importance of hand reaching to sound as a guiding tool that provides additional motor interaction with the sound; this could explain the significant decrease in the head reaction times at the end of the spatial training. The reaching-to-sound procedure can also maximize head movement accuracy and, therefore, accelerates spatial learning in this population [16,18].

### 4.3. Clinical Relevance of Motion-Tracked Virtual Reality in Treating Auditory Deficit

This novel technique permits continuous tracking of the head and body interaction with sound and allows us to control different factors involved in auditory behavior that are difficult to evaluate in classical methods [14].

Using this approach, we can deliver a wide variety of stimulus types at different spatial positions in the free field. A free field sound emission allows us to target different types of hearing impairments, including patients with hearing aids and cochlear implants. In addition to the ability of the device to control multiple factors involved in auditory behavior, the device is user-friendly and accessible to different age ranges, including children, which makes its utility in clinics more relevant.

In the present design, we aimed to verify the feasibility of audiovisual training in the spatial rehabilitation of UHL. Auditory spatial behavior improved after one session of spatial training; however, the present protocol can be optimized in order to maintain a long-term benefit and improve spatial perception in daily life. In future studies, we could increase the number of training sessions; although one session showed an effect on spatial behavior that lasted even after the non-spatial training (group 1), the frequency of the training can be an important factor in consolidating spatial gain. Studies on monaural-plugged ferrets showed quick and extensive improvement in spatial performance when spatial training was provided regularly in daily sessions. In the study of [15] on monaurally plugged normal hearing controls the number of sessions of spatial audiovisual training was five training sessions during one week (one session per day). A recent study by Coudert et al. [22] on bilateral cochlear implants showed that intensive spatial training of eight sessions over 10 weeks significantly improved spatial performance and also performance in the speech in noise, which was evaluated by a matrix test, while four sessions spread over 2 weeks seemed to be insufficient. Such results indicate that the number of sessions and the duration between each session are important factors for the efficiency of a training program. All of the mentioned studies that used VR showed its clinical relevance in developing training programs for patients with hearing deficits. Thus, to eliminate the possibility of device habituation that might have an effect on behavior, an evaluation of spatial performance outside of the VR before and after training can be useful. This could help explain, for example, the significant reduction of head-pointing errors for participants number 15 and number 19 during the washout phase. To eliminate such a possibility in the future, and in order to control the VR habituation effect, a control condition of a sound localization task outside of VR is useful. In addition, spatial testing in VR can be ameliorated by adding natural audiovisual presentations, such as the use of realistic, immersive scenes with more realistic sounds.

## 5. Conclusions

Unilateral hearing loss disrupts binaural integration and alters spatial function. However, adaptation to monaural listening conditions can be enhanced and accelerated by a regular audiovisual training program, allowing for active listening in motion-tracked virtual reality devices, which can become clinically useful tools that control all possible factors involved in spatial auditory behavior.

## Figures and Tables

**Table 1:** Demographic information about the 19 unilateral hearing loss patients.

ID	Gender	Age	D. Onset	Side of Deafness	Etiology of Deafness
UHL 01	M	37	5	R	Sudden
UHL 02	M	20	16	R	Congenital
UHL 03	F	43	12	R	Granulome
UHL 04	F	56	7	L	Facial paralysia
UHL 05	F	55	21	L	Unknown
UHL 06	M	44	2	R	Unknown
UHL 07	F	47	8	R	Sudden
UHL 08	F	70	64	L	Tympanic lesion
UHL 09	M	60	13	L	Unknown
UHL 10	F	65	5	L	Vestibular schwanoma
UHL 11	M	69	3	L	Vestibular schwanoma
UHL 12	F	56	3	L	Vestibular schwanoma
UHL 13	F	59	4	L	Congenital
UHL 14	M	48	10	L	Neurinoma
UHL 15	M	55	5	R	Vestibular schwanoma
UHL 16	M	55	11	R	Intracochlear schwanoma
UHL 17	F	47	8	R	Unknown
UHL 18	F	59	59	L	Congenital
UHL 19	F	43	1	R	Cholestéatome

D. Onset for Deafness Onset, F for Female, M for Male, L for Left, and R for Right. White lines correspond to participants belonging to group 1, and gray lines correspond to participants belonging to group 2.

## Data Availability

The data are not publicly available due to the quality of personal health data containing information that could compromise the privacy of research participants. Given the nature of the data and in order to comply with the Sponsor’s data protection policy, a data-sharing agreement must be established with the Toulouse University Hospital before the access.
